# Acute Respiratory Failure Due to Airway Obstruction in Forestier Syndrome: A Case Report

**DOI:** 10.7759/cureus.80259

**Published:** 2025-03-08

**Authors:** Marta Magno, Carolina Roriz, Cátia Santos, Nuno Ferreira, Ana Araújo

**Affiliations:** 1 Critical Care Medicine, Unidade Local de Saúde da Região de Leiria, Leiria, PRT

**Keywords:** airway management, airway obstruction, dysphagia, forestier’s syndrome, respiratory failure

## Abstract

Diffuse idiopathic skeletal hyperostosis (DISH) is characterized by calcification and ossification of ligaments, predominantly affecting the spine. Although often asymptomatic, it can cause dysphagia, dyspnea, and airway obstruction when anterior cervical osteophytes are involved. A 70-year-old male presented with severe dysphagia, respiratory distress, and weight loss. Magnetic resonance imaging (MRI) revealed prominent anterior cervical osteophytes (C2-C7), causing airway narrowing and esophageal compression, consistent with DISH. Acute respiratory failure with stridor required urgent intubation and mechanical ventilation. Due to surgical limitations at the initial hospital, the patient was transferred to a tertiary central facility for specialized care. This case highlights the diagnostic and therapeutic complexities of DISH-related airway obstruction. Multidisciplinary care proved critical, including corticosteroids, mechanical ventilation, and nutritional support. Acute respiratory decompensation may have been triggered by epiglottitis secondary to microaspiration. Early recognition and coordinated multidisciplinary management are essential for achieving optimal outcomes in severe DISH cases involving airway obstruction.

## Introduction

Forestier syndrome or diffuse idiopathic skeletal hyperostosis (DISH) is a condition characterized by osteophyte formation due to calcification of ligaments and tendons [1,2]. Throughout the spine, this condition is most frequently observed in the thoracic and lumbar vertebrae, while it is less common in the cervical region [3]. Cervical osteophytosis occurs in 12%-30% of the general population and is usually asymptomatic [4]. The most commonly involved segment is between C4 and C7 [5]. Anterior spinal osteophytes may protrude into the posterior pharyngeal space leading to anterior displacement of the larynx and potentially causing dysphonia, dysphagia, dyspnea, and, in severe cases, acute airway obstruction [6]. The severity of the disease and the extent of ossification can vary significantly [7]. Although the exact etiology remains unclear, metabolic disorders such as obesity, hyperlipidemia, diabetes, and hypertension have been identified as potential risk factors [1,7]. This report discusses the complex management of a 70-year-old male presenting with severe dysphagia, respiratory distress, and significant weight loss due to prominent anterior cervical osteophytes. The case highlights the acute management strategies as well as the diagnostic and therapeutic challenges associated with this condition.

## Case presentation

We report the case of a 70-year-old male, who presented to the emergency department due to acute onset of dyspnea, a squeaky cough, hoarseness, odynophagia for the past 12 hours, associated with progressive dysphagia, initially for solids and then for liquids, with a history of approximately six months, resulting in a 15kg weight loss (current weight was 73kg, and his current body mass index was 21.1kg/m^2^). As a personal history of hypertension and hyperlipidemia in treatment with candesartan and rosuvastatin. He did not have any preexisting allergies. Symptomatology at admission improved after medical treatment with bronchodilators and intravenous corticosteroid therapy. Upon admission, the patient also exhibited respiratory acidosis, requiring the initiation of non-invasive mechanical ventilation. The patient was admitted for further investigation and care.

**Figure 1:**
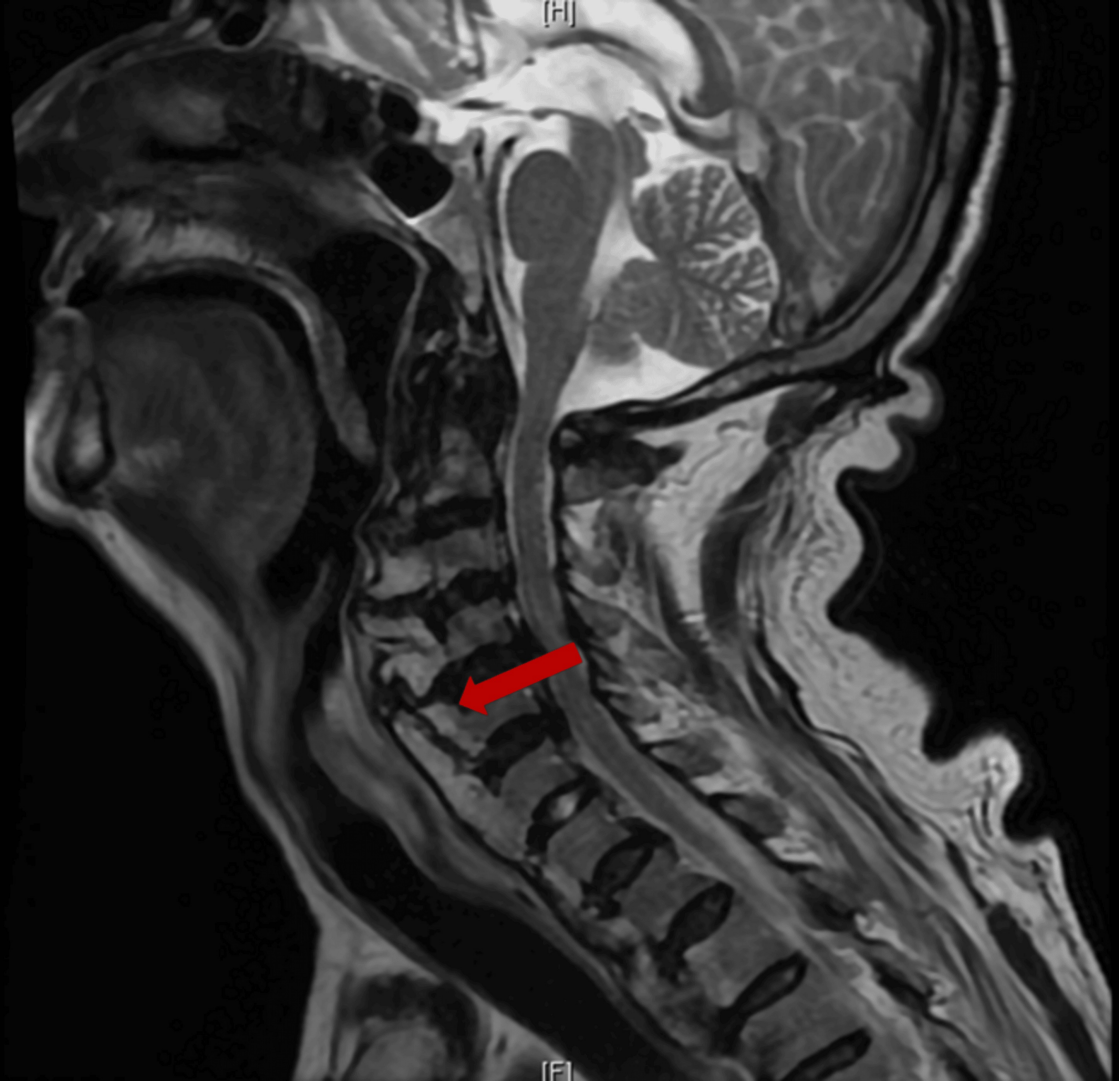
MR imaging showing exuberant anterior osteophyte causing significant upper airway narrowing and esophagus compression (red arrow)

During hospitalization, a multidisciplinary approach was undertaken to determine the underlying cause of his condition. Gastroenterology evaluation revealed oedema and congestion of the hypopharyngeal mucosa, leading to the placement of a nasogastric tube for enteral feeding. Otorhinolaryngology assessment identified signs of reflux, including retrocricoid oedema and laryngeal penetration, but no evidence of structural injury or impaired laryngeal mobility. Neurological causes were ruled out following a neurological assessment. The patient underwent a full diagnostic work-up to determine the underlying cause of his clinical presentation. This included a magnetic resonance imaging (MRI), which revealed prominent anterior cervical osteophytes extending from C2 to C7 (Figure 1), causing significant upper airway narrowing and esophageal compression.

On the 21st day of hospitalization, the Emergency Team was activated due to severe respiratory failure with stridor. Clinical assessment indicated the need for orotracheal intubation and mechanical ventilation due to extrinsic airway obstruction leading to respiratory exhaustion. A rapid sequence intubation (RSI) was performed using fentanyl (100mcg), propofol (60mg), ketamine (100mg) and rocuronium (90mg), calculated for an estimated weight of 75kg. Airway management was successfully achieved with videolaryngoscopy, and a 7.5mm orotracheal tube was placed at 22cm without complications. The patient's respiratory status improved, and supplemental oxygen was no longer required. Given the surgical and anesthetic limitations of the community hospital, the patient was stabilized and transferred to the tertiary care center under the care of the Neurosurgery team for further treatment.

## Discussion

DISH, first described by Forestier and Rotes Querol in 1950 [8], is a non-inflammatory disorder that primarily affects men in their mid-60s [5,9], with an estimated prevalence of 42% in individuals over 65 years of age [10]. DISH is characterized by ossification of the anterior longitudinal ligament, paravertebral osteophyte formation, and ligamentous calcification [11,12].

The diagnosis is based on Resnick’s radiological criteria, which include (I) Osseous bridging of at least four contiguous vertebral bodies; (II) absence of degenerative disc disease, and (III) absence of inflammatory changes in facets and sacroiliac joints.

Our case involves a male patient in his 70s with a history of hypertension and dyslipidemia, comorbidities frequently reported in the literature [13]. While dysphagia in DISH typically develops gradually, this patient presented acutely with respiratory compromise. The proposed mechanisms of dysphagia in DISH include mechanical compression, periesopharyngeal inﬂammation with edema and ﬁbrosis due to chronic irritation, and cricopharyngeal spasm also due to chronic irritation [10,12]. Retrospective analysis suggests that the acute presentation may have been triggered by epiglottitis secondary to microaspiration caused by progressive dysphagia. This highlights the importance of early recognition and multidisciplinary approach. Treatment strategies depend on the severity of the symptoms. Mild to moderate cases are managed conservatively with nonsteroidal anti-inflammatory drugs, muscle relaxants, and dietary modifications. Surgical intervention is reserved for severe cases involving progressive dysphagia, significant weight loss, airway obstruction, disabling dysphonia, or when conservative measures fail [11,14].

This case illustrates the challenges of managing airway obstruction in DISH in an acute setting. The literature suggests that awake fibreoptic intubation is the safest approach [7]. However, in this case, due to the urgency of the situation and the patient's respiratory failure, videolaryngoscopy along with RSI, was selected as the initial approach given the patient’s acute respiratory failure and the risk of further decompensation, the loss of spontaneous ventilation was justified by the need for immediate control over the airway. As a contingency plan, an emergency cricothyrotomy was considered, with input from anesthesiology ensuring that all potential complications were anticipated and multidisciplinary teams ready to perform. There are reports of emergency tracheostomy being required in similar cases, but this was not an available option in our hospital [7].

## Conclusions

Cervical osteophytosis, although typically asymptomatic, can be a potential cause of dysphagia and acute airway obstruction. Recognition of this condition is crucial for prompt diagnosis and appropriate management. This case emphasizes the critical role of intensive care in managing respiratory failure resulting from extrinsic airway obstruction. Coordinated multidisciplinary discussions and interventions are key to achieving the best outcomes for the patient.

## References

[1] Soares D, Bernardes F, Silva M, Miradouro J, Lopes D (2023). Diffuse idiopathic skeletal hyperostosis (dish)-phagia: a case report and review of literature of a rare disease manifestation. Cureus.

[2] Nelson RS, Urquhart AC, Faciszewski T (2006). Diffuse idiopathic skeletal hyperostosis: a rare cause of dysphagia, airway obstruction, and dysphonia. J Am Coll Surg.

[3] Iida M, Tanabe K, Dohi S, Iida H (2015). Airway management for patients with ossification of the anterior longitudinal ligament of the cervical spine. JA Clin Rep.

[4] Gosavi K, Dey P, Swami S (2018). Airway management in case of diffuse idiopathic skeletal hyperostosis. Asian J Neurosurg.

[5] Gokce A, Beyzadeoglu T, Hanci L, Erdogan F (2007). Diffuse idiopathic skeletal hyperostosis as a cause of acute respiratory distress in early postoperative period of total knee arthroplasty. Arch Orthop Trauma Surg.

[6] Caminos CB, Cenoz IZ, Louis CJ, Otano TB, Esáin BF, Pérez de Ciriza MT (2008). Forestier disease: an unusual cause of upper airway obstruction. Am J Emerg Med.

[7] Thompson C, Moga R, Crosby ET (2010). Failed videolaryngoscope intubation in a patient with diffuse idiopathic skeletal hyperostosis and spinal cord injury. Can J Anaesth.

[8] Zamorano SG, Manzano SGV, Artal AA, Ángel PR, Herreros NG (2019). Acute airway obstruction in a patient with forestier disease. Case report (Article in Spanish). Rev Esp Anestesiol Reanim.

[9] Karaarslan N, Gürbüz MS, Çalışkan T, Simsek AT (2017). Forestier syndrome presenting with dysphagia: case report of a rare presentation. J Spine Surg.

[10] Sunakawa J, Yano H, Kinjo M (2023). Dysphagia with diffuse idiopathic skeletal hyperostosis. BMJ Case Rep.

[11] Giammalva GR, Iacopino DG, Graziano F, Gulì C, Pino MA, Maugeri R (2018). Clinical and radiological features of Forestier's disease presenting with dysphagia. Surg Neurol Int.

[12] Marks B, Schober E, Swoboda H (1998). Diffuse idiopathic skeletal hyperostosis causing obstructing laryngeal edema. Eur Arch Otorhinolaryngol.

[13] Kiss C, Szilágyi M, Paksy A, Poór G (2002). Risk factors for diffuse idiopathic skeletal hyperostosis: a case-control study. Rheumatology (Oxford).

[14] Di Martino A, Costa V, Denaro V (2006). Dysphagia and dysphonia due to anterior cervical osteophytes: report of a patient affected by DISH. Eur J Orthopaed Surgery Traumatol.

